# Computer-Assisted Recovery of Threatened Plants: Keys for Breaking Seed Dormancy of *Eryngium viviparum*

**DOI:** 10.3389/fpls.2017.02092

**Published:** 2017-12-12

**Authors:** Manuel Ayuso, Pablo Ramil-Rego, Mariana Landin, Pedro P. Gallego, M. Esther Barreal

**Affiliations:** ^1^Applied Plant and Soil Biology, Faculty of Biology, University of Vigo, Vigo, Spain; ^2^GI-1934 TB-Biodiversity, IBADER, Universidad de Santiago, Lugo, Spain; ^3^Department of Pharmacology, Pharmacy and Pharmaceutical Technology, Faculty of Pharmacy, University of Santiago de Compostela, Santiago de Compostela, Spain

**Keywords:** Apiaceae, artificial intelligence, conservation, germination, dormancy, endemic plant, embryo seed ratio, underdeveloped embryo

## Abstract

Many endangered plants such as *Eryngium viviparum* (Apiaceae) present a poor germination rate. This fact could be due to intrinsic and extrinsic seed variability influencing germination and dormancy of seeds. The objective of this study is to better understand the physiological mechanism of seed latency and, through artificial intelligence models, to determine the factors that stimulate germination rates of *E. viviparum* seeds. This description could be essential to prevent the disappearance of endangered plants. Germination *in vitro* was carried out under different dormancy breaking and incubation procedures. Percentages of germination, viability and E:S ratio were calculated and seeds were dissected at the end of each assay to describe embryo development. The database obtained was modeled using neurofuzzy logic technology. We have found that the most of *Eryngium* seeds (62.6%) were non-viable seeds (fully empty or without embryos). Excluding those, we have established the germination conditions to break seed dormancy that allow obtaining a real germination rate of 100%. Advantageously, the best conditions pointed out by neurofuzzy logic model for embryo growth were the combination of 1 mg L^−1^ GA_3_ (Gibberellic Acid) and high incubation temperature and for germination the combination of long incubation and short warm stratification periods. Our results suggest that *E. viviparum seeds* present morphophysiological dormancy, which reduce the rate of germination. The knowledge provided by the neurofuzzy logic model makes possible not just break the physiological component of dormancy, but stimulate the embryo development increasing the rate of germination. Undoubtedly, the strategy developed in this work can be useful to recover other endangered plants by improving their germination rate and uniformity favoring their *ex vitro* conservation.

## Introduction

*Eryngium viviparum* is a small biennial aquatic plant that belongs to Apiaceae family, endemic to the European Atlantic region and with distribution in NW France, NW Portugal and NW Spain (Romero et al., 2004). Their natural habitat is flat areas subjected to seasonal flooding, living submerged for 7–9 months of the year. Aquatic plants are one of the most threatened groups in the European flora, mainly due to anthropic habitat alteration and destruction (Romero et al., 2004; Ramil-Rego and Dominguez-Conde, 2006; Magnanon et al., 2012). In 1997, *E. viviparum* was classified as *vulnerable* by International Union for Conservation of Nature (IUCN) and included in red list of threatened plants (Walter and Gillett, 1998). Moreover, *E. viviparum* has been listed in Annex I of the Berne Convention and considered by Directive 92/43/EEC as a priority species (Annex II). Its classification has recently been changed to *endangered* due to the loss of many subpopulations, the drastic reduction of the area of occupancy (<80 km^2^ in the world) and the decrease in the quality of its habitat and of the number of individuals (Lansdown, 2011).

The main threats for endangered plants are human pressure over the territory, competition with other species, overgrazing and collecting that cause degradation, fragmentation, and massive reduction of their natural habitats (Ramil-Rego and Dominguez-Conde, 2006; González-Benito and Martín, 2011; Lansdown, 2011; Magnanon et al., 2012). Although high protection and access restrictions to their natural habitats have been implemented, for *in situ* conservation, the situation is critical for some species as *E. viviparum*. New *ex situ* conservation strategies, such as *in vitro* propagation are needed and should be urgently designed and implemented in order to protect them. Different micropropagation procedures have been developed for endangered and endemic plants in the past 30 years and, also for this particular species (Ba-ares et al., 2004; González-Benito and Martín, 2011). The critical population size of *E. viviparum* does not allow sufficient material from its natural habitat for micropropagation. Therefore, Fay (1992) has proposed its seeds as the material of choice for *ex situ* conservation, which allows preserving their biodiversity.

Many species of Apiaceae family are well known by a non-uniform and asynchronized seed germination (Mozumder and Hossain, 2013), which promotes germination rates lower than 10% and limits their *in situ* conservation. Moreover, low germination in Apiaceae is due to several kind of dormancy state in seeds and by the presence of high amount of non-viable seeds (Robinson, 1954; Ojala, 1985). Different strategies have been developed to overcome dormancy problems in several Apiaceae, including cold and/or warm stratification (Necajeva and Ievinsh, 2013), the use of plant growth regulators such as gibberellic acid (GA_3_) and kinetin (KIN) (Mozumder and Hossain, 2013) or the incubation temperature (Finch-Savage and Leubner-Metzger, 2006). As far as we know no studies have addressed the low germination rate problem in *E. viviparum*, which remains a challenge.

In recent years, several artificial intelligence tools have been applied to model and predict the effect of different variables in plant tissue culture (Zielinska and Kepczynska, 2013), monitoring seed growth and vigor (Chaugule, 2012) or testing germination (Dell'Aquila, 2004). In this work, neurofuzzy logic has been applied to investigate the cause-effect relationships between several germination factors (dormancy breaking stratification and germination conditions) and seed germination responses (percentage of germination, embryo seed rate, etc.). Neurofuzzy logic is a hybrid approach that combines the adaptive learning capabilities from artificial neural networks with the generality of representation from fuzzy logic through simple “IF-THEN” rules. This methodology has been previously and successfully used as advanced decision support tool (Gallego et al., 2011; Gago et al., 2014).

On this purpose, this study attempts to get insight on the germination of *E. viviparum* seeds and the causes of its low germination rate. Neurofuzzy logic is applied to model germination results as a function of several germination conditions in order to find the key factors that control or stimulate the embryo development, facilitating the completion of germination. Understanding the physiological mechanism responsible for seed germination should help to design new procedures for any other endangered plant with low germination rates.

## Materials and methods

### Plant material

Fruits (schizocarps) of *E. viviparum* Gay were collected on the margin of the Cospeito Lake, Lugo, Spain (43°14′30.16″N, 7° 32′55.539″W) in September 2013 and 2014. Harvested mature brown fruits were kept in dry paper bags under room laboratory conditions until used. Individual mericarps (seeds) were obtained by mechanical friction and stored dry in plastic Petri dishes at 4°C for 12 weeks in darkness.

### Germination conditions

*Eryngium viviparum* Gay is an endangered plant and its use is legally limited, being extremely difficult to obtain both fruits and/or seeds, therefore two batches of just 750 seeds (2013) and 1,440 seeds (2014) were used in this study. The initial experiments were adjusted to maximize the number of seeds per treatment (55–100), whereas in the following experiments the treatments were performed with 80 seeds each (see Table 1).

**Table 1:** Design of *Eryngium* seed dormancy-breaking and incubation procedures.

		**Dormancy-breaking**				**Incubation**				
**Treatment**	**Seeds (N°)**	**Strat. 4°C (Weeks)**	**Strat. 25°C (Weeks)**	**Moisture condition**	**GA_3_ (mg L^−1^)**	**Substrate**	**Temp (°C)**	**GA_3_ (mg L^−1^)**	**KIN (mg L^−1^)**	**Time (weeks)**
1	95	8	4	Dry	0	MS	24	0	0	20
2	95	8	4	Dry	0	MS	24	1	0	20
3	95	8	4	Dry	0	MS	24	0	1	20
4	55	8	4	Dry	0	MS	18-13	0	0	20
5	55	8	4	Dry	0	MS	18-13	1	0	20
6	55	8	4	Dry	0	MS	18-13	0	1	20
7	100	8	24	Dry	0	MS	18-13	0	0	20
8	100	8	24	Dry	0	MS	18-13	0	1	20
9	100	8	24	Dry	0	MS	18-13	0	5	20
10	80	0	4	Dry	0	Water	18-13	0	0	10
11	80	0	4	Dry	0	MS	18-13	0	0	10
12	80	0	4	Dry	0	MS	18-13	0	1	10
13	80	0	4	Dry	0	MS	18-13	0	5	10
14	80	0	4	Wet	0	Water	18-13	0	0	10
15	80	0	4	Wet	0	MS	18-13	0	0	10
16	80	0	4	Wet	0	MS	18-13	0	1	10
17	80	0	4	Wet	0	MS	18-13	0	5	10
18	80	0	4	Wet	2	Water	18-13	0	0	10
19	80	0	4	Wet	2	MS	18-13	0	0	10
20	80	0	4	Wet	2	MS	18-13	0	1	10
21	80	0	4	Wet	2	MS	18-13	0	5	10
22	80	8	0	Wet	0	Water	18-13	0	0	10
23	80	8	0	Wet	0	MS	18-13	0	0	10
24	80	8	0	Wet	0	MS	18-13	0	1	10
25	80	8	0	Wet	2	Water	18-13	0	0	10
26	80	8	0	Wet	2	MS	18-13	0	0	10
27	80	8	0	Wet	2	MS	18-13	0	1	10

*Seed were previously stored in darkness at 4°C during 12 weeks (MS, Murashige and Skoog medium; GA_3_, gibberellic acid, and KIN, kinetin)*.

Several treatments have been carried out to improve *Eryngium* germination including seed surface sterilization and stratification (cold and warm), to stimulate seed dormancy breaking and to induce embryo growth and development.

### Seed sterilization

Seeds were surface sterilized before (wet stratification) or after (dry stratification) of the dormancy-breaking treatments.

In both cases, seeds were soaked in 2% sodium hypochlorite for 5 min. After, in laminar flow cabinet, seeds were washed with sterile distilled water for three times, and stirred in 50% sulfuric acid for 40 min. Seeds were removed from sulfuric solution, washed again for three times during 5 min, and soaked overnight in sterile distilled water, previous dormancy breaking and incubation experiments.

### Dormancy-breaking procedure

On the first batch of seeds, long periods of cold stratification (12 + 8 weeks at 4°C) followed by a variable period of warm stratification (4–24 weeks at 25°C) were tested. All seeds (750) were stratified in dry paper filter in Petri dishes (treatments 1–9; Table 1).

Further, with the second batch of seeds, also shorter periods of cold stratification (12 + 0) and warm stratification (0–4 weeks at 25°C) than in 2013 were tested (treatments 10–13). Wet treatments (water or 2 mg L^−1^ GA_3_ solutions) were also included (treatments 14–27). In these treatments, seeds were surface sterilized previously.

### Incubation procedure

Once completed dormancy breaking procedures, surface sterilized seeds were sowed in sterilized glass culture vessels on 25 mL MS medium (Murashige and Skoog, 1962) supplemented with GA_3_ (0 and 1 mg L^−1^) or KIN (0, 1, and 5 mg L^−1^) or in plastic Petri dishes on double filter paper layer moistened with 20 mL sterile distilled water (Table 1). Sterilization was carried in autoclave at 121°C for 20 min at 105 kPa.

Incubation was carried out in growth chambers at 24 or 18–13°C thermoperiod (similar to the habitat temperature) and 12/12 h photoperiod (Flux density of 55 μmol m^−2^ s^−1^) during 10 and 20 weeks. All combinations are shown in Table 1.

### Germination test

Germinated and no-germinated seed percentage was calculated after 20 or 10 weeks (Table 1) treatments 1–9 and 10–27, respectively. Seeds with longer than 1 mm visible radicle were considered as germinated. No-germinated seeds were dissected by its longitudinal axis and evaluated using a stereomicroscope (Nikon SMZ-U). In order to measure and calculate the embryo and seed length ratio (E:S ratio; Vandelook et al., 2007a), the camera software package (0.7X DXM Lens Nikon) was used.

Another batch of seeds (50 in 2013 and 2014) were stored during 12 weeks in darkness at 4°C, dissected and their ratio E:S calculated in order to know the embryo development in the moment of start the germination treatments. This E:S ratio was used as control.

Once germinated or dissected, seeds were split out into two groups: viable and non-viable seeds. All germinated seeds and those no germinated but with embryo visible were included as “viable seed” and employed to calculate the following parameters:

- ***Seed germination percentage*****: %G** = (N° germinated seeds/N° seeds) ^*^ 100
- ***Real seed germination percentage*****: %RG** = (N° germinated seeds/N° viable seeds) ^*^ 100
- ***No germinated seeds percentage*****: %NG** = (N° no germinated viable seeds/N° seeds) ^*^ 100
- ***E:S ratio*** = Embryo length/Seed length. Germinated seeds were considered as E:S = 1 and included in the calculation average E:S ratio.

Non-germinated seeds were considered as “non-viable,” including embryoless seeds and those completely empty.

Two new parameters were calculated for the non-viable seeds:

- ***Embryoless seeds percentage*****: %EL** = (total N° embryoless seeds/total N° seeds) ^*^ 100
- ***Empty seeds percentage*****: %EM** = (total N° empty seeds/total N° seeds) ^*^ 100

### Statistical analysis

Student *t*-test was performed to test the significant difference (*P* < 0.05) of E:S ratio respect to control.

### Neurofuzzy logic

A database with results from 27 treatments was modeled using the commercial neurofuzzy logic software, FormRules v4.03 (Intelligensys Ltd., UK). Neurofuzzy logic combines the learning capabilities of neural networks with the linguistic capabilities of fuzzy logic. Neurofuzzy model allows to model germination results as a function of the several factors studied and predict results for a not study combination of factors. Additionally, it allows expressing the model through simple IF…THEN rules providing understanding and knowledge. Every IF-THEN rule is associated to a membership degree which represents the degree of truth from 0 to 1 (Gallego et al., 2011; Gago et al., 2014; Nezami-Alanagh et al., 2014, 2017).

For the modeling, the inputs were the nine germination variables studied during dormancy breaking (time of stratification at 4°C, time of stratification at 25°C, moisture condition, and GA_3_ concentration) and incubation (substrate, temperature, GA_3_ concentration, KIN concentration and time) and the outputs were the six germination parameters defined above (%G, %NG, %EL, %EM, %RG, and E:S ratio).

A separate model was developed for each output. The parameters used for modeling by FormRules® are shown in Table 2.

**Table 2:** The training parameters setting with FormRules v3.31.

**MINIMIZATION PARAMETERS**
Ridge regression factor: 1 e^−6^
**MODEL SELECTION CRITERIA**
Structural Risk Minimization (SRM)
C1 = 0.8–0.854 C2 = 4.8
Number of set densities: 2
Set densities: 2. 3
Adapt nodes: TRUE
Max. inputs per SubModel: 4
Max. nodes per input: 15

## Results

### Seed sterilization

Field sampled *E. viviparum* seeds presented severe contamination, which was not eliminated by the procedures described for other Apiaceae. Despite of several disinfection procedures as ethanol 70% and sodium hypochlorite 1–2%, *Eryngium* seeds remained highly contaminated (60–100%). Additional strong treatments including seed soak in sodium hypochlorite 2% for 5 min, long rise in distilled water and stir in 50% sulfuric acid for 40 min were necessary to completely eliminate contamination.

### Seed germination

Table 3 presents *E. viviparum* germination percentages (%G) for all the conditions studied. As it can be seen, the germination values are really poor for all the treatments; being the maximum seed germination percentage 27.3% for treatment 2. The highest germination values were reached on treatments 2, 3, and 8 (>20%G), which shared the next conditions: long cold dry stratification period (12 + 8 weeks) without GA_3_ and long incubation of 20 weeks (Table 1). Treatments 18, 19, 20, 21, and 25, give no germination at all (Table 3). Seeds on these treatments were stratified in wet conditions (with 2 mg L^−1^ GA_3_) and thermoperiod for 10 weeks (Table 1).

**Table 3:** Percentage of germination (%G), no germination (%NG), embryoless seeds (%EL), empty seeds (%EM), viable (%G+%NG), non-viable (%EL+%EM) seeds, real germination (%RG), and mean and standard error of the E:S ratio.

**Treatment**	**%G**	**%NG**	**%EL**	**%EM**	**%Viable**	**%Non-viable**	**%RG**	**E:S ratio (± SE)**
1	7.1	14.3	28.6	50.0	21.4	78.6	33.3	0.51 (± 0.25)
2	**27.3**	**0.0**	45.5	27.3	27.3	72.7	**100.0**	1.00 (± 0.00)^*^
3	**22.2**	33.3	**0.0**	44.4	55.6	44.4	40.0	0.50 (± 0.21)
4	17.8	2.2	11.1	68.9	20.0	80.0	88.9	0.98 (± 0.02)^*^
5	12.8	10.3	23.1	53.8	23.1	76.9	55.6	0.71 (± 0.13)^*^
6	16.0	12.0	8.0	64.0	28.0	72.0	57.1	0.74 (± 0.14)^*^
7	13.6	27.3	8.0	51.1	40.9	59.1	33.3	0.54 (± 0.06)^*^
8	**22.2**	20.8	15.3	41.7	43.1	56.9	51.6	0.68 (± 0.06)^*^
9	15.1	19.2	19.2	46.6	34.2	65.8	44.0	0.62 (± 0.07)^*^
10	5.4	39.5	34.0	21.1	44.9	55.1	12.1	0.48 (± 0.05)^*^
11	3.3	36.7	34.0	26.0	40.0	60.0	8.3	0.47 (± 0.05)^*^
12	1.3	34.2	**36.2**	28.2	35.6	64.4	3.8	0.43 (± 0.04)^*^
13	4.0	28.7	20.7	46.7	32.7	67.3	12.2	0.57 (± 0.08)^*^
14	2.1	37.6	27.0	33.3	39.7	60.3	5.4	0.43 (± 0.04)^*^
15	5.0	43.3	22.7	29.1	48.2	51.8	10.3	0.45 (± 0.04)^*^
16	4.8	32.9	29.5	32.9	37.7	62.3	12.7	0.49 (± 0.06)^*^
17	2.8	40.0	27.6	29.7	42.8	57.2	6.5	0.42 (± 0.04)^*^
18	0.0	35.7	32.2	32.2	35.7	64.3	0.0	0.36 (± 0.02)
19	0.0	38.5	29.4	32.2	38.5	61.5	0.0	0.33 (± 0.01)
20	0.0	42.8	26.9	30.3	42.8	57.2	0.0	0.35 (± 0.01)
21	0.0	42.4	32.6	25.0	42.4	57.6	0.0	0.36 (± 0.01)^*^
22	2.3	32.6	28.0	37.1	34.9	65.1	6.6	0.45 (± 0.06)^*^
23	1.7	28.2	31.6	38.5	29.9	70.1	5.8	0.49 (± 0.08)^*^
24	1.1	39.5	29.4	29.9	40.7	59.3	2.8	0.37 (± 0.03)
25	0.0	41.7	30.3	28.0	41.7	58.3	0.0	0.37 (± 0.01)^*^
26	1.7	41.0	28.3	28.9	42.8	57.2	4.1	0.40 (± 0.04)^*^
27	1.1	45.4	30.5	23.0	46.6	53.4	2.5	0.37 (± 0.03)
Mean	**7.1**	**30.4**	**25.5**	**37.0**	**37.4**	**62.6**	**22.1**	**0.51 (± 0.03)**

Treatment with E:S different significantly with control (0.31) at p < 0.05 is indicated (^*^).

**Figure 1:**
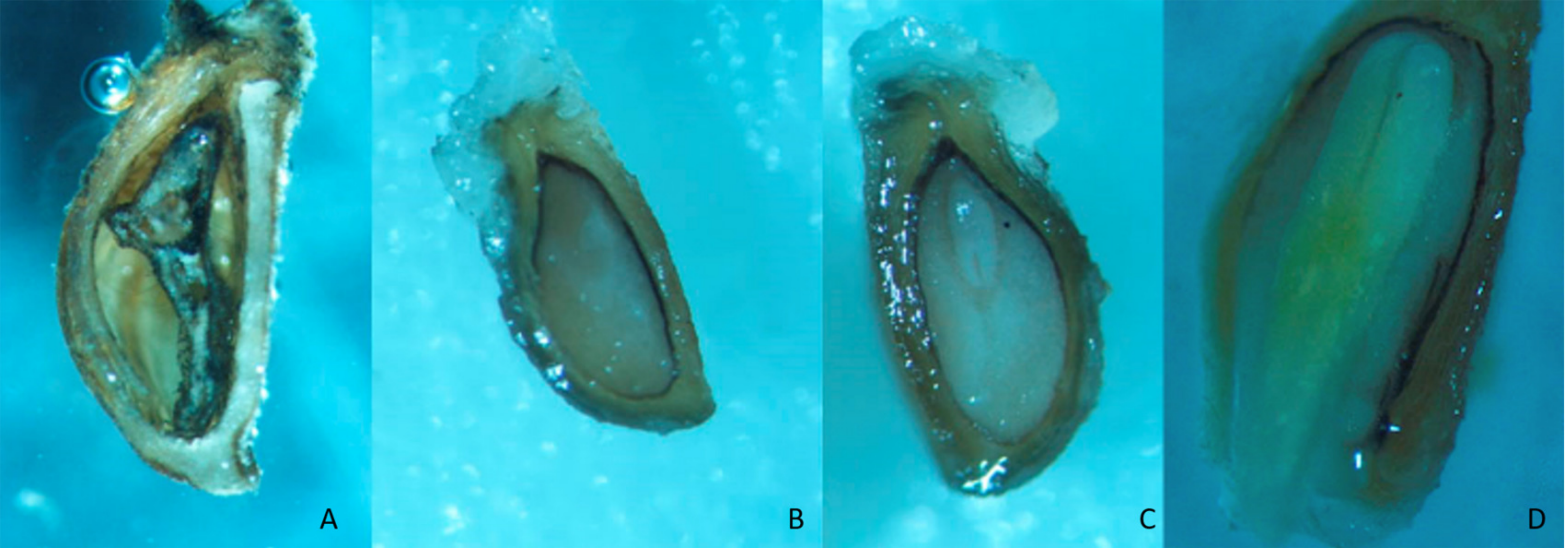
Dissected *Eryngium* seeds empty **(A)**, embryoless **(B)**, with underdeveloped embryo **(C)** and fully developed embryo **(D)**.

Looking for the reasons for those low germination rates, no germinated seeds were dissected. As it can be seen in Figure 1, the images show that seeds could be classified as empty (A), embryoless (B), seeds with underdeveloped embryo (C), and with fully developed embryo (D).

Percentages of no germinated seeds (%NG), embryoless (%EL), and empty seeds (%EM) help to explain previous germination percentages (Table 3). The overall low germination rate was due to a high number of NG seeds (mean 30.4%) and non-viable seeds (mean 62.6%), from which 25.5% where embryoless and 37.0% empty seeds, respectively (Table 3). These results suggest that, only a maximum of 37.4% could be germinated in optimal conditions (7.1 %G + 30.4 %NG).

If the germination percentage is recalculated considering just the viable seeds, named as real seed germination (%RG; Materials and Methods) the results are promising, as some of the treatments reach 89–100% (treatments 2 and 4; Table 3).

Table 3 also shows the E:S ratio obtained at the end of each treatment. All treatments have values higher than 0.31 (control E:S ratio from seeds stored during 12 weeks at 4°C) although not significant differences were found for some of the treatments against control (Table 3). This results point out the important role of some of the conditions used during dormancy–breaking and incubation procedures on embryo development and justifies the modeling in order to understand which and how they affect germination.

Neurofuzzy logic succeeded in simultaneously modeling the six germination parameters. Table 4 shows the inputs that explain the variability of each output together with the quality parameters for the models obtained: correlation coefficients (*R*^2^) and ANOVA parameters (calculated *f* ratio, degrees of freedom and *f* critical for α = 0.01). As it can be seen *R*^2^-values ranged from 74.85 to 95.92% indicating high model predictabilities. Moreover, ANOVA *f*-ratio values were always higher than the corresponding f critical values (α < 0.01) indicating their accuracy. It is interesting to note several general features from these results: (a) Only 6 out of 9 inputs help to explain the variability of the outputs studied, (b) The factor time of incubation has been highlighted by the model as important for all the parameters, (c) %RG and E:S are explained by the same set of inputs including two from dormancy breaking phase (Strat. 25°C and GA_3_) and three from incubation (Time, GA_3_ and Temp), and (d) The variation of the parameters %NG, %EL, and %EM, which are associated with low percentages of germination, are explained by the time of incubation and the concentration of GA_3_ during the incubation period, but also involves other variables such as the time of stratification at 25°C or the temperature and the concentration of KIN during incubation.

**Table 4:** Critical factors from the neurofuzzy logic Train *R*^2^ and ANOVA parameters for training [*f* ratio, degree of freedom (df1: model and df2: total) and *f* critical value for α < 0.01] for each output.

**Outputs**	**Critical factors**	**Train set *R*^2^**	***f* ratio**	**df1, df2**	***f* critical**
%G	**Time**	79.69	47.0883	2, 26	5.53
%NG	**Time** GA_3_ (I)	74.85	22.8146	3, 26	4.64
%EL	**Time** Temp × KIN GA_3_ (I)	85.97	20.4282	6, 26	3.59
%EM	**Time** Temp Strat. 25°C GA_3_ (I)	81.32	18.278	5, 26	5.80
%RG	**Strat. 25°C× Time** Temp × GA_3_ (I) GA_3_ (D-B)	95.92	52.8306	8, 26	3.29
E:S	**Temp × GA_3_ (I)** Time Strat. 25°C GA_3_ (D-B)	90.39	25.5405	7, 26	3.42

*Concentration of GA_3_ during dormancy breaking (D-B) or incubation (I) period. The inputs with stronger effect on each output have been highlighted*.

IF-THEN rules generated by the neurofuzzy logic software with an associated membership degree are shown in Table 5. All factors included in the models were fuzzyficated as low or high indicating a linear effect on germination parameters in the range of the study, independently if they have a significant effect alone or in interaction with other factor. Those rules greatly simplify the process of understanding the cause-effect relationship among factors and germination parameters. As an example, the percentage of germination variability is explained by just one factor: time of incubation (Table 4). IF time of incubation is low, THEN the %G will be low (Rule 1; Table 5). The membership 0.93 indicates that in this condition the percentage of germinated *Eryngium* seeds predicted by the model will be close to the lowest % G values in the database. Therefore, 20 weeks of incubation promotes higher germination percentages than 10 weeks, as it is for treatments 2, 3, and 8 with the highest %G (highlighted in Table 3).

**Table 5:** Rules generated by neurofuzzy logic.

**Rules**		**Strat. 25°C**	**GA_3_ (D-B)**	**Temp**	**GA_3_ (I)**	**KIN**	**Time**		**G**	**NG**	**EL**	**EM**	**RG**	**E:S**	**Membership degree**
**1**	**IF**						**Low**	**THEN**	**Low**						**0.93**
**2**							**High**		**High**						**0.63**
3	**IF**						**Low**	**THEN**		**High**					0.90
4							**High**			**Low**					0.95
5					Low					High					0.77
6					High					Low					0.82
**7**	**IF**						**Low**	**THEN**			**High**				**0.54**
**8**							**High**				**Low**				**1.00**
9					Low						Low				1.00
10					High						Low				0.61
11				Low		Low					High				1.00
12				Low		High					High				1.00
**13**				**High**		**Low**					**High**				**1.00**
**14**				**High**		**High**					**Low**				**1.00**
**15**	**IF**						**Low**	**THEN**				**Low**			**1.00**
**16**							**High**					**High**			**1.00**
17				Low								High			0.89
18				High								Low			1.00
19		Low										High			0.98
20		High										Low			1.00
21					Low							High			0.67
22					High							Low			1.00
**23**	**IF**	**Low**					**Low**	**THEN**					**Low**		**1.00**
24		High					Low						Low		1.00
**25**		**Low**					**High**						**High**		**1.00**
26		High					High						Low		0.59
27				Low	Low								Low		0.61
28				High	Low								Low		1.00
29				Low	High								Low		1.00
30				HIgh	High								High		1.00
31			Low										Low		0.51
32			High										Low		0.71
33	**IF**						Low	**THEN**						Low	1.00
34							High							High	1.00
35				Low	Low									High	0.60
**36**				**High**	**Low**									**Low**	**1.00**
37				Low	High									Low	1.00
**38**				**High**	**High**									**High**	**1.00**
39		Low												High	0.96
40		High												Low	1.00
41			Low											Low	0.54
42			High											Low	1.00

*The inputs with the highest membership degree (stronger effect) on each output indicates by the model are highlighted*.

On the contrary, the %NG variability is explained by two factors: time and concentration of GA_3_ during the incubation period (Table 4). Neurofuzzy logic highlights the key role of incubation time but logically, in the opposite way than for %G (Table 5): if low incubation time is used, the percentage of no germination for *Eryngium* seeds is high (rule 3; membership 0.90) and if the time of incubation is high then %NG is low (rule 4; 0.95). In the same sense if the plant growth regulator GA_3_ is present in the incubation solution (1 mgL^−1^) then the %NG will be low. These rules explain for example, the high value for %G together with low value for %NG obtained for treatment 2 (Table 3).

Two kinds of non-viable seeds were detected: embryoless but with endosperm (EL; Figure 1B) or without endosperm (EM; Figure 1A). The percentage of both in *Eryngium* seeds is determined by incubation time, but in a different way (Table 5). While a long period of incubation promotes always low embryoless seeds percentages (rule 8; membership 1.0), also promotes high percentages of empty seeds (rule 16; 1.0). The interaction of high temperature and high kinetin concentration gives low embryoless seeds percentages (rule 14; 1.0), as can be seen when compared treatments 3 (24°C) vs. 12 (18–13°C) in Table 3. The presence (high) of GA_3_ during incubation (rules 10 and 22) also reduces the %EL and %EM, in agreement with the results obtained for %NG (rule 6).

The percentage of real seed germination (RG) variability can be explained by a selection of five inputs (the interaction of time of stratification at 25°C and time of incubation, the interaction of temperature and concentration of GA_3_ during incubation and the concentration of GA_3_ during dormancy breaking process). From the rules corresponding to RG parameter (Table 5) it is easy to deduce that; (a) a short period of warm stratification (low) combined to a long period of incubation (high weeks) rendered, always, high %RG (rule 25; membership 1.0); (b) high temperature in combination with 1 mg L^−1^ GA_3_ during incubation also promotes high % real germination (rule 30; 1.0); and (c) the addition of GA_3_ during dormancy breaking period, fully inhibits *Eryngium* seed germination (rules 31–32). All this conditions can be observed in treatment 2 (Table 1) with high %RG (100%; Table 3).

High E:S ratio favor seed germination. Neurofuzzy pointed out, that the interaction high incubation temperature and 1 mg L^−1^ GA_3_ promotes embryo growth (rule 38; membership 1.0) as in treatment 2 (Table 3). Long periods of incubation (rule 34; 1.0) together with short warm stratification periods (rule 39; 0.96) improve E:S (Table 5). On the contrary, the presence of GA_3_ in the wet solution during the break dormancy procedure reduce E:S ratio (rule 42; 1.0). These results are in fully agreement with those described for %RG.

## Discussion

According to literature review, no disinfection or general antiseptic procedures such as ethanol 70% for 30 s followed by sodium hypochlorite at low concentration 1% for 5–10 min were enough for surface seed sterilization in Apiaceae family (Walmsley and Davy, 1997; Vandelook et al., 2008; Thiem et al., 2013). Those typical procedures did not work at all for *E. viviparum* seeds. All seeds revealed severe contamination by fungi during germination and none were able to germinate. Only after application of strong antiseptic procedures including sulfuric acid, a method commonly used to surface-sterilize seeds heavily contaminated with fungi (Latchs and Christensen, 1985; Siegel et al., 1987), we succeeded in achieving the total elimination of fungal contamination.

Poor germination rate is a common problem in the Apiaceae family (Robinson, 1954; Ojala, 1985; Baskin and Baskin, 2014). *Eryngium* genus is not an exception. *Eryngium maritimum* (Walmsley and Davy, 1997; Necajeva and Ievinsh, 2013), *Eryngium planum* (Thiem et al., 2013) and *Eryngium foetidum* (Mozumder et al., 2011; Mozumder and Hossain, 2013), have shown lower than 20, 12, and 10% germination respectively, without dormancy breaking treatments. Little information on *Eryngium viviparum* is available. Our results ranging from 0 to 27% of germination are in agreement with Magnanon and coworkers that reported low germination percentages (10–40%) on populations from France and Spain (Magnanon et al., 2012).

Low germination rates in the Apiaceae family have been correlated with the next three causes (Robinson, 1954; Ojala, 1985; Baskin and Baskin, 2014): (1) presence of high percentage of non-viable seeds without embryo; (2) presence of high percentage of seed with underdeveloped embryos; and (3) presence of dormant seeds.

In agreement with the first cause our results showed a high non-viable seeds percentage (62.5%) close to other Apiaceae as *Anethum graveolens* (ranged 39–62%; Robinson, 1954) or *Anthriscus caucalis* (49%; Rawnsley et al., 2002). Among the potential causes for non-viable seeds (zygote degeneration, death of embryo, mutations, etc.; Baskin and Baskin, 2014), two have been described for Apiaceae family: (1) insect infestation; these seeds have an endosperm but not embryo (Flemion and Henrickson, 1949) and (2) self-pollinated umbels; seeds produced in these umbels are usually empty (without endosperm and embryo; Ojala, 1985). In this work, the high percentage of non-viable seeds have been described as embryoless (25.5% EL) and empty (37% EM), supporting the hypothesis that the poor germination percentage of *E. viviparum* seeds can be explained as a consequence of the high percentage of non-viable seeds due to insect infestation and self-pollinated umbels.

The second cause of low germination in Apiaceae is the presence of underdeveloped embryos at the moment of dispersal (Martin, 1946). The embryo needs to grow up to a critical length before germination. E:S ratio was investigated to check the presence of underdeveloped embryos in *E. viviparum* (Table 3). The E:S ratio seeds stored during 12 weeks at 4°C in darkness, used as control, averaged 0.31, similar to those values found for other Apiaceae species as *Torilis japonica* with a ratio 0.25, *Angelica sylvestris* with 0.29, or *Selinum carvifolia* with 0.31 at harvest. However, after 20 weeks at 5°C, those embryos growth until 0.29, 0.39, and 0.34, respectively, a little higher than the E:S ratio at harvest (Vandelook et al., 2007a, 2008). This delay in seed germination due to the underdeveloped and differentiated embryo is called morphology dormancy (MD, Nikolaeva, 1977), and those Apiaceae has been characterized as MD seed (Vandelook et al., 2007a,b, 2008). Our results, clearly demonstrated a high percentage 30.4% of underdeveloped embryo seeds suggesting that *E. viviparum* also showed MD dormancy, in agreement with other *Eryngium* sp. (Necajeva and Ievinsh, 2013). Seeds with MD, as Apiaceae, need specific conditions as a moist substrate, suitable temperature and photoperiod as growth embryo requirements (Baskin and Baskin, 2014). If seeds remain under these conditions, embryos should growth and germinate in 4 weeks or less. No precise protocol to overcome this problem (the need of right conditions enabling embryo growth and development until reach a critical size to germinate) has been previously described in the literature for *E. viviparum*.

The third cause for low germination rates in Apiaceae is the existence of an additional physiological mechanism of dormancy, which also inhibits germination (Baskin and Baskin, 2004). Some Apiaceae present both dormancies morphological (MD) and physiological (PD) at the same time, known as morphophysiological dormancy (MPD), and need a considerably longer period for germinate than MD seeds (Baskin and Baskin, 2004). The combination of cold and warm stratification can be effective for break MPD dormancy and allow the embryo to growth. Warm stratification was effective in some species of *Osmorhiza* and *Erythronium* genus (Baskin et al., 1995). In addition, short warm stratification increases the seed sensitivity to GA_3_ improving germination rate in *E. maritimum* and suggesting a key role of GA_3_ to overcome the dormancy (Vandelook et al., 2008; Necajeva and Ievinsh, 2013). *E. viviparum* presents low percentage of seeds (mean 7.1%) that germinate after breaking the MPD. Interesting, some treatment, such as 2, reached 27.3%G and 100%RG, which means that all viable seeds were able to break MPD dormancy and germinate.

To understand the physiological mechanism of dormancy breaking, a representative study of seed germination require test several factors, which implies many different treatments and replicates, and finally large batches of seeds. Those requirements are essential for an accurate interpretation of the cause-effect of those factors and their interactions on seed germination (International Seed Testing Association, 1985). As stated above, *E. viviparum* is an endangered species and therefore, no large seed batches are available. Based on these limitations, two experiments including just 27 treatments were proposed. We test the most important factors well known as dormancy breaking in Apiaceae such as cold and warm stratification and two PGR (GA_3_ and KIN) well known as inducers of embryo development (Vandelook et al., 2008; Mozumder and Hossain, 2013; Necajeva and Ievinsh, 2013). The authors are aware that the design space was not well-sampled, which hinders a conventional statistical treatment to analyse the results and draw conclusions. Neural networks has demonstrated to be a practical approach to deciphering the key factors in several biological process and an excellent alternative to conventional statistical methods (Gago et al., 2010a,b; Gallego et al., 2011; Nezami-Alanagh et al., 2014; Arab et al., 2016). Advantageously, neurofuzzy logic technology allows working with not well-defined design spaces and different kind data at the same time (Nezami-Alanagh et al., 2017). In this paper, that technology was used to reduce the number of treatments and therefore, the needed seeds, without losing key information to extract scientific conclusions.

Neurofuzzy logic succeeded in modeling all outputs simultaneously with high predictability and accuracy (Table 3). Additionally, it allowed obtaining a set of rules that explain the cause-effect among the factors (inputs) and the germination parameters obtained (Table 4). The model pinpointed that the factor time of incubation was the most significant factor *for Eryngium* seed germination; long periods (around 20 weeks) are strongly recommended to obtain high %G, low %NG, low %EL, high E:S, and high %RG, but in this case in combination with low warm stratification. In fact only after 20 weeks of incubation the highest real germination rates can be reached (see treatment 2 and 4; Table 3). Interesting, also with high incubation periods a high percentage of empty seeds was obtained, suggesting a high correlation between endosperm degradation and duration of the germination period. The model also identified the important role of the interaction between incubation temperature and GA_3_ concentration. If temperature is around 24°C and 1 mg L^−1^ GA_3_ is present, the highest real germination and E:S was obtained. The third most important factor was the factor stratification at 25°C. Low period of warm stratification (4 weeks) is preferred to obtain the highest % of real germination and E:S ratio (1.00 membership). Some authors (Necajeva and Ievinsh, 2013) demonstrated that seed of *E. maritimum* seed germinated to a high final germination percentage only after 12–16 weeks cold stratification. Our model suggests that *E. viviparum* presents seeds with MDP, and needs to break dormancy. Long incubation time (20 weeks) combined with low warm stratification (4 weeks) at continuous high temperature 24°C and 1 mg L^−1^ GA_3_ are strongly recommended.

## Author contributions

MA: Performed the experiments; PR-R: Contributed with reagents/materials; ML and PG: Contributed modeling/analysis tools; PG and MB: Conceived and designed the experiments. All authors contributed to writing of the manuscript.

### Conflict of interest statement

The authors declare that the research was conducted in the absence of any commercial or financial relationships that could be construed as a potential conflict of interest.
